# Electrochemical Detection of Ascorbic Acid in Finger-Actuated Microfluidic Chip

**DOI:** 10.3390/mi13091479

**Published:** 2022-09-06

**Authors:** Xing Liu, Mi Li, Jiahui Zheng, Xiaoling Zhang, Junyi Zeng, Yanjian Liao, Jian Chen, Jun Yang, Xiaolin Zheng, Ning Hu

**Affiliations:** 1 Laboratory of Biorheological Science and Technology, Ministry of Education, Bioengineering College, Chongqing University, Chongqing 400030, China; 2 School of Smart Health, Chongqing College of Electronic Engineering, Chongqing 401331, China; 3 Center for Drug Evaluation & Inspection of Chongqing Municipal Drug Administration, Chongqing 401120, China

**Keywords:** electrochemical sensor, ascorbic acid, 3D printing, microfluidic chip, micropump

## Abstract

The traditional quantitative analysis methods of ascorbic acid (AA), which require expensive equipment, a large amount of samples and professional technicians, are usually complex and time-consuming. A low-cost and high-efficiency AA detection device is reported in this work. It integrates a three-electrode sensor module prepared by screen printing technology, and a microfluidic chip with a finger-actuated micropump peeled from the liquid-crystal display (LCD) 3D printing resin molds. The AA detection process on this device is easy to operate. On-chip detection has been demonstrated to be 2.48 times more sensitive than off-chip detection and requires only a microliter-scale sample volume, which is much smaller than that required in traditional electrochemical methods. Experiments show that the sample and buffer can be fully mixed in the microchannel, which is consistent with the numerical simulation results wherein the mixing efficiency is greater than 90%. Commercially available tablets and beverages are also tested, and the result shows the reliability and accuracy of the device, demonstrating its broad application prospects in the field of point-of-care testing (POCT).

## 1. Introduction

Ascorbic acid (AA), or vitamin C, is a water-soluble vitamin with acidity and strong reducibility, which is widely found in fruits, vegetables and organisms [1]. It is a highly effective antioxidant that can inhibit the oxidative damage of free radicals to the human body and prevent the occurrence of tumors and cancers [2]. It participates in redox reactions in the human body and plays an important role in the metabolism of folic acid, some amino acids and hormones. It can also promote the absorption of inorganic iron elements [3,4]. Considering the potential influence of the immune response and reactive oxygen, it has been hypothesized to be useful for the prevention or treatment of COVID-19 [5]. AA is an essential compound to maintain human health, but it cannot be synthesized by the human body and needs to be obtained from food and medicine [2]. Meanwhile, the AA content affects the ripening of some fruits, because the oxidant/antioxidant balance affects the ripening time [6]. Therefore, quantitative analysis of AA in foods, medicine and the human body is an important topic in the field of biomedicine.

Commonly used methods for the quantitative analysis of AA include absorbance photometry, high-performance liquid chromatography, electrochemical methods, titrimetric methods [7], fluorescence spectroscopy [8], chemiluminescence [9], enzymatic methods [10], capillary electrophoresis [11], etc. Among them, electrochemical technology is widely used because of its advantages such as convenience, low cost, miniaturization and high dynamic range [12,13,14,15]. One of the most frequently used electrochemical analysis methods for the quantitative detection of AA is voltammetry [16], which does not require high accuracy of equipment, technician capabilities, complicated procedures or sample pretreatment to provide low detection limits. The electrochemical analysis methods are usually based on a three-electrode system, which includes a main electrode, a reference electrode and an auxiliary electrode. Common types of electrodes include glassy carbon electrodes, screen-printed electrodes, metal electrodes, calomel electrodes and various modified electrodes developed by researchers [14,17,18,19,20,21,22,23]. Screen-printed electrodes (SPE) have attracted high attention due to the advantages of being low-cost and easy to mass produce, and they do not require a complex surface treatment process [23,24,25]. For example, Alonso-Lomillo et al. used a sensor based on gold nanoparticles and SPCE to constitute a simple methodology, accurate and economical for the amperometric detection of AA, but the sensor needed to be immersed in 5 mL of supporting electrolyte [26]. There is a need to develop a convenient integrated device for the real-time, low-cost and rapid on-site detection of samples. For example, the rapid sampling and testing of drugs and beverages is required to prevent substandard products from entering the market. On-chip electrochemical detection combined with SPE and microfluidics is a suitable option.

Recently, microfluidic technology has promoted the development of on-chip detection, which can realize small volumes of sample, rapid sample injection, mixing and reaction in a small-sized chip [27,28,29,30,31]. Various types of micropumps have been developed to replace external pumping systems, which are bulky, complex and expensive [32,33]. In addition, some researchers focus their work on micromixers, which ensure high-efficiency sample mixing and high-accuracy detection. In these works, a polydimethylsiloxane (PDMS) microfluidic chip based on soft lithography [34,35] is a conventional technology due to its high optical transparency and deformability [36,37,38]. Additionally, soft lithography can be used to obtain micrometer-to-nanometer-level features, but it requires stringent and expensive experimental conditions. Moreover, 3D printing has shown the advantages of being low-cost, efficient and enabling high-aspect-ratio microfluidic constructions in recent years [39,40], representing a new microfluidic chip fabrication method. However, 3D printing still has problems of lower resolution and lower optical transparency [41,42].

In this work, by combining the advantages of SPE-based electrochemical detection methods and microfluidic technology, a high-efficiency and easily fabricated AA detection device is developed. It includes a three-electrode sensor module and a PDMS microfluidic chip with a finger-actuated hybrid micromanipulator. The three-electrode sensor is prepared by screen printing technology. The PDMS hybrid micromanipulator with a micropump and micro-mixture is peeled from the resin mold, which is printed by an LCD 3D printer. Typically, milliliter volumes of sample and supporting electrolyte are added to the same vessel and mixed with a stirrer or an oscillator. In this work, the design of the mixing channel enables the microliter-scale volume of the sample to be thoroughly mixed with the supporting electrolyte rapidly, without the need for external equipment.

The sensor shows good mixing performance and higher detection sensitivity than the off-chip method on a microfluidic chip integrated with a finger-actuated micropump, and it requires simple operation, low costs and a smaller sample volume, which illustrates its potential to be applied to the POCT of physiological fluids, medicine and food.

## 2. Materials and Methods

### 2.1. Materials, Reagents and Equipment

Conductive carbon ink (423S) was provided by Atchison, and conductive silver ink (YT-981C) was provided by Yituo (Suzhou, China). The AA analytical reagent and the phosphate buffer solution (PBS, pH 7.4) were purchased from SolarBio (Beijing, China), and deionized (DI) water was collected from the ULUPURE (Sichuan, China) instrument. Polydimethylsiloxane (PDMS) and a curing agent were purchased from Dow Corning (Midland, TX, USA). The molds were silanized with Trichloro (1H,1H,2H,2H-perfluorooctyl) silane (TPFS) purchased from Acmec (Shanghai, China). UV-curable resin molds were printed by Anycubic Photon M3 Plus (Shenzhen, China). The PDMS parts were bonded after being treated in a plasma cleaner purchased from Fangrui Technology (Shenzhen, China). Electrochemical measurements were carried out using a three-electrode system connected to the electrochemical workstation CHI660E purchased from CH Instruments (Shanghai, China). An impedance analyzer (SI-1260, Solartron Analytical, Farnborough, UK) was used for EIS measurement. ZView software (Scribner Associates Inc., Southern Pines, NC, USA) and Origin 9.0 (origin, Inc., Northampton, MA, USA) were used for data fitting. Simulations were performed by COMSOL Multiphysics (Stockholm, Sweden).

### 2.2. Preparation of Electrochemical Sensor Chip

**Figure 1:**
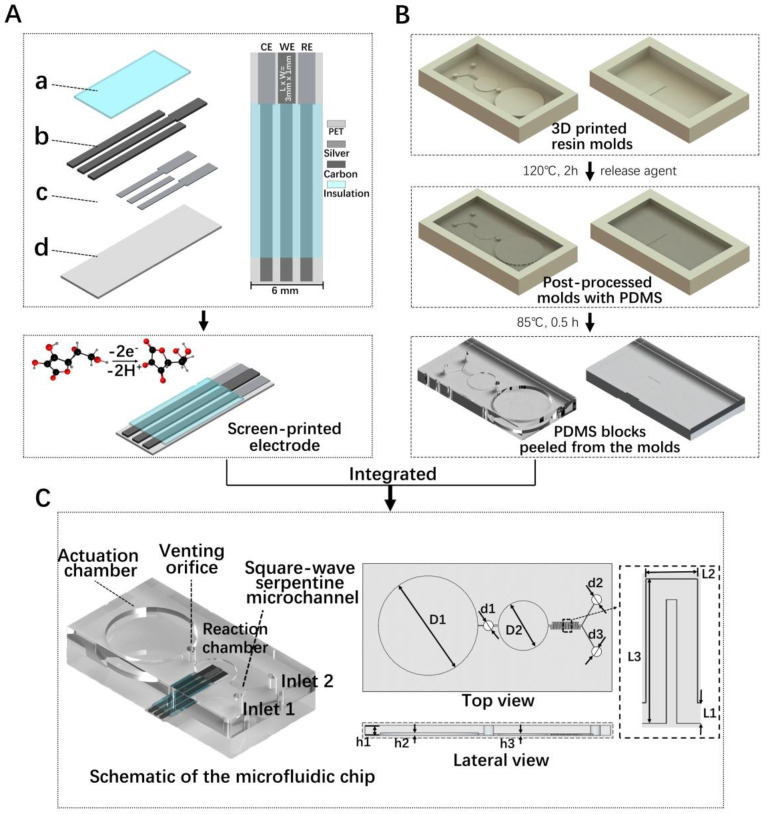
(**A**) Scheme of silver/carbon screen-printed electrode (SPE). (a) Insulating layer, (b) carbon track/electrode, (c) silver track/electrode and (d) polyethylene terephthalate (PET) substrate. (**B**) Scheme of 3D-printed molds and PDMS blocks. (**C**) Schematic of the integrated microfluidic chip and additional details (reference values: d1 = d2 = d3 = 1–1.5 mm, D1 = 20 mm, D2 = 10 mm, L1 = 0.2 mm, L2 = 0.3 mm, L3 = 1.2 mm, h1 = 1.5–3 mm, h2 = 0.5 mm, h3 = 0.2 mm).

Figure 1A shows the manufacturing process of the SPEs. A conventional three-electrode system was employed, including mixtures of graphite and carbon black as the working electrode (WE), and commercial silver material as the counter electrode (CE) and reference electrode (RE). The frames with a screen mesh for the designed pattern were manufactured, including a PET substrate layer, silver conductive layer/electrode layer, carbon conductive layer/electrode layer and insulating paint layer. A 300-mesh polyester screen with a tension of 20 N was selected for printing, and the photosensitive film thickness was 12–15 μm; a 75° polyurethane squeegee was suitable. Firstly, a layer of silver ink was printed on a 0.35-mm-thick PET substrate and then thermally cured in an oven at 80 °C for 20 min (referring to the ink recommendations for thermal curing temperature and time). Then, approximately 8-μm-thick carbon ink was overprinted on it and thermally cured. Finally, two 8-μm layers of insulating ink were printed and thermally cured.

The geometry and the dimensions of the microfluidic chip, which included a micromixer and a micropump, as shown in Figure 1C, were designed. Among a variety of reported hybrid structures, a micromixer with square-wave serpentine microchannels [43] has been applied in this chip, which has a channel width of 100 μm and a total length of 25.2 mm. A micropump with a cylindrical designed actuation chamber allows users to easily operate it with a single finger. It is worth noting that if h1 is much higher than h2 (h1−h2 > 3 mm), a large pressure needs to be applied. However, if h1 is not high enough (h1−h2 < 1 mm), the PDMS does not have enough resilience after pressing. Considering that the pressing operation step may damage the electrode surface, the driving chamber and the reaction chamber were separated.

Besides the advantage of low costs, 3D printing technology allows us to fabricate different height structures in one step, which avoids complex and time-consuming multiple lithography procedures, and the alignment operation. As mentioned above, the horizontal structural dimensions of the chip were 100 μm to 5 mm, while the vertical direction structural dimensions were 200 μm to 3 mm. It is easy to fabricate by the 3D printing technology directly. In this work, the microchannel molds were prepared by 3D-printed photocurable resin instead of traditional lithography. The PDMS hybrid micromanipulator fabrication process is shown in Figure 1B. Firstly, the chip model was imported into the software for slicing, and the printing parameters were set as follows: 1.5 s for the UV projection time of each layer and 0.02 mm for the thickness of each slice. The printing process can be completed in 45 min. The model was removed and cleaned in 75% alcohol for 10 min, and then the model was UV post-cured for 60 min.

Since PDMS is difficult to be polymerized into a solid on the surface of 3D-printed materials, the mold needs to be post-processed [44]. According to the reference [45], post-treatment procedures include heating, plasma cleaning, surface silanization and other methods, among which heating is the most important. Therefore, we heated the mold on a hot plate at 120 °C for 120 min, and then cleaned it in a plasma cleaner for 3 min, and then spin-coated its surface with 200 µL trichloro (1H,1H,2H,2H-perfluoro-octyl) silane to prevent the mold from sticking to the PDMS. After vacuum degassing for 2 h, PDMS was poured onto the mold and heated at 85 °C for 30 min in an oven. Two PDMS parts were successfully stripped, punched and bonded. Finally, the electrode was inserted into the reserved PDMS groove.

### 2.3. Sample Preparation and Electrochemical Methods

The pure AA powder was dissolved to obtain a 25,000 μM AA solution, and then diluted to 5000 μM, 2500 μM, 1000 μM, 500 μM, 250 μM, 200 μM, 150 μM, 100 μM, 50 μM and 10 μM successively and stored in cold storage away from light. The detection methods were cyclic voltammetry (CV), differential pulse voltammetry (DPV) and electrochemical impedance spectroscopy (EIS).

## 3. Results and Discussion

### 3.1. Characterization and Electrochemical Behavior of SPEs

**Figure 2:**
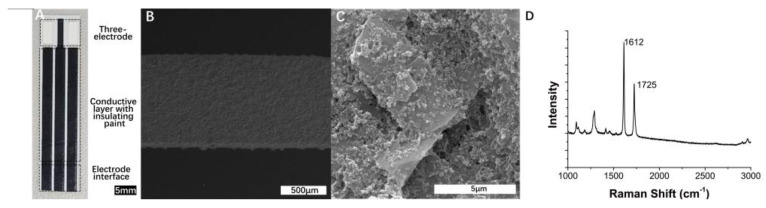
Images of SPE and SEM micrographs of the carbon electrode. (**A**) Overall structure morphology of the SPE. (**B**,**C**) Low-magnification and high-magnification SEM images of the carbon electrode. (**D**) Raman spectrum of carbon electrode.

Figure 2A is the overall structure morphology of the fabricated SPE. The microstructure and morphology of the SPEC were characterized by scanning electron microscopy (SEM). Figure 2B shows that the surface morphology of the SPEC was relatively uniform and compact. Moreover, Figure 2C shows that a large number of carbon black nanoparticles were distributed on the surface of the graphite. From the Raman spectrum at an excitation wavelength of 532 nm in Figure 2D, the positions of the observed bands at 1612 cm^−^^1^ and 1725 cm^−^^1^ are in agreement with the Raman bands reported previously for carbon black [46]. Such carbon black particles increased the surface roughness of the electrodes and therefore increased the specific surface area, which is beneficial for constructing a highly dispersed conductive network and enhancing the electrochemical signal [47,48].

**Figure 3:**
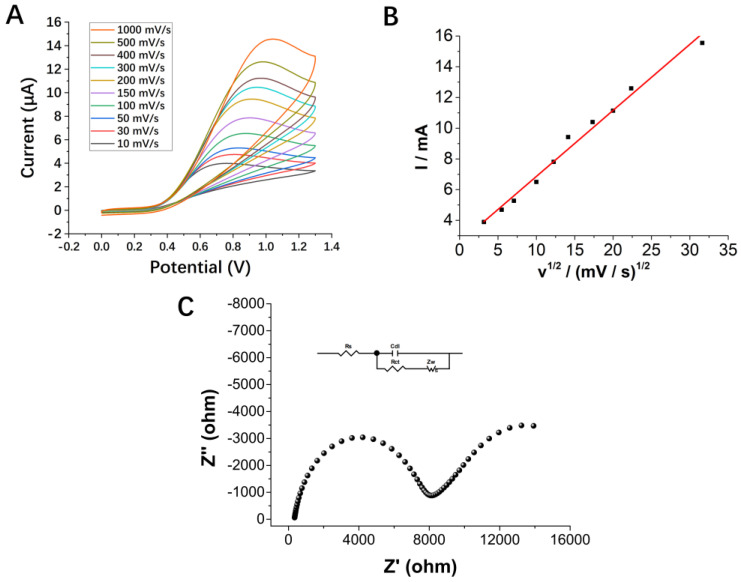
(**A**) CV responses of SPE at different scan rates (10–1000 mV/s) in 2500 µM of AA solution. (**B**) The plot of the anodic current versus the square root of increasing scan rates (10–1000 mV/s), R^2^ = 0.98597. The experiments were carried out at a volume of 40 μL sample on the electrode surface. (**C**) Nyquist plots in the frequency range from 0.01 Hz to 100 kHz. Inset is Randles equivalent circuit.

We first evaluated the electrochemical performance of the fabricated SPEs under an off-chip detection system to confirm their detection performance and reproducibility. To approximate the condition of on-chip detection, 40 μL of the sample was dropped on the electrode surface, instead of conducting the experiment in a traditional reaction vessel. Figure 3A displays the cyclic voltammograms (CVs) of the electrode between 0 V and 1.3 V, at different scan rates from 10 mV/s to 1000 mV/s, in 2500 µM of AA solution. Figure 3B shows that the anodic peak currents were proportional to the square root of the scan rate ((V/s)1/2) from 10 to 1000 mV/s, and no obvious reduction peak was observed during reverse scanning. The results revealed that the reaction was controlled by the diffusion of species and it was an irreversible surface-controlled process of electron transfer [49,50]. In addition, the electron transfer behavior between the electrolyte and the electrode material was studied by electrochemical impedance spectroscopy (EIS). The impedance spectrum was fitted with ZView software as a Randles circuit, shown as an inset in Figure 3C; Rs, Rct, Cdl and Zw correspond to solution resistance, charge transfer resistance, double-layer capacitance and Warburg impedance, respectively. The semicircle in the high-frequency region describes the electron transfer process, and its diameter is equal to the charge transfer resistance (Rct), which has a value of 8.160 kΩ.

**Figure 4:**
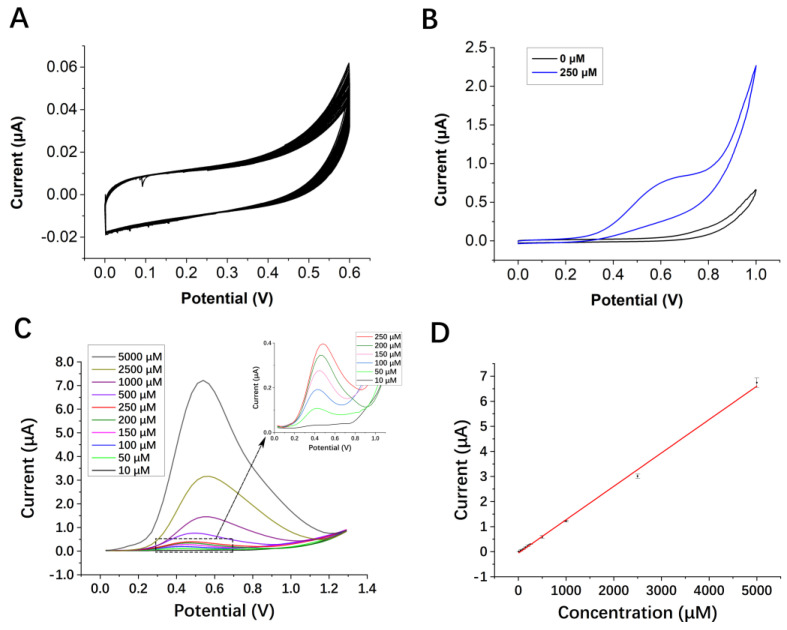
(**A**) The 50 sweep segments CV curve in PBS at a scan rate of 50 mV s^−1^. (**B**) Comparative DPV responses of SPE with 250 µM and without AA. (**C**) DPV responses of SPE with increasing concentration of AA (10–5000 µM), and inset is a partial enlargement of 10–250 µM (*n* = 3). (**D**) The calibration plot of current vs. concentration of AA (*n* = 3).

The CV study was carried out to preliminarily explore the oxidation behavior of the electrode when AA was added. Figure 4A shows the 50 sweep segments CV curve in PBS, which proves the robustness of the electrode [51]. Figure 4B is a comparison of the CV curves obtained at a scan rate of 50 mV s^−1^. Compared with the electrolyte without the addition of AA, the current increases in the presence of 250 µM AA, which indicates that AA was oxidized during the reaction. The DPV was used to analyze different concentrations of AA, as seen in Figure 4C. Compared with CV, the DPV method can provide a single-run voltammetric detection with better sensitivity and efficiency [52]. Figure 4D shows the DPV response fitting curve of the electrode in different concentrations of AA solution. The results showed that as the concentration of AA increases, the peak oxidation current increases linearly. It indicates that the concentration of the analyte is proportional to the peak oxidation current. AA is a substance with a lactone ring, which undergoes two-electron oxidation to generate dehydroascorbic acid. This linear relationship confirms that the analyte releases 2e^−^ and 2H^+^ during the oxidation process, which in turn increases the oxidation peak current [50]. In the CV pre-test shown in Figure 4B, the scan rate is set to 0.05 V/s. According to Figure 3A, the higher scan rate leads to stronger polarization and larger oxidation peak potential. After optimization, the DVP detection parameters are finally determined as follows: initial E = 0 V, final E = 1.3 V, step E = 0.03 V, amplitude = 0.04 V. As shown in Figure 4D, when *n* = 3 (three different electrodes of the same batch), there was an obvious linear relationship between the peak current and the concentration of AA. It has a calculated detection limit value of 1.038 µM and a linear range of 10 µM–5000 µM. Separately, twenty SPEs in the same batch were used to detect the same concentration of AA, and the relative standard deviation (RSD) was 8.95%, indicating the good reproducibility of the SPEs. Variation caused by different production batches can be resolved by normalization.

**Figure 5:**
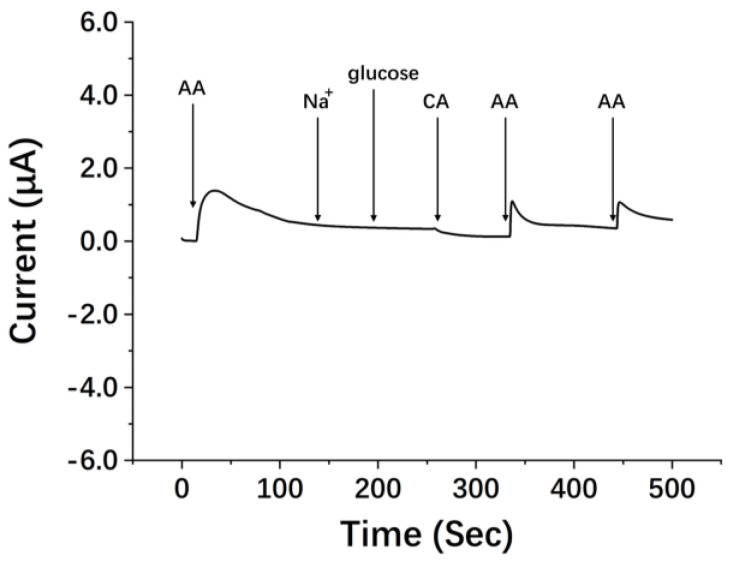
Amperometric response of SPE to successive addition of 250 µM of AA, Na^+^ ions (250 µM), glucose (1 mM), CA (250 µM) and then AA (250 µM) twice at a potential of +0.4 V.

In addition, selectivity is a very challenging issue for non-enzymatic detection methods. Therefore, when the oxidation potential was +0.4 V, the influence of interfering substances such as, Na^+^ ions, glucose and citric acid (CA) on the SPEs’ detection of AA was tested by the amperometric method. As shown in Figure 5, 250 μM UA, 250 μM Na^+^ ions, 1 mM glucose and 250 μM CA had little interference with the detection of 250 μM AA, which indicated the selectivity and reliability of the sensor. We also supplemented the results of voltammetry under the same conditions. The raw data of DPV detection in the presence of interfering substances are shown in Supplementary Material Figure S9 (without noise reduction). It can be seen from the figure that the interfering substances have little effect on AA detection, and there is only a slight fluctuation at 0.9 V.

### 3.2. The Evaluation of Finger-Actuated Micropump and On-Chip Detection

The serpentine channel was modeled and numerically simulated. The model utilizes the Laminar Flow and Transport of Diluted Species interfaces to analyze the flow field and concentration distribution. The flow rate at the inlet is roughly 10^−^^9^ m^3^/s and a low Reynolds number of 0.005 is set for a water solution. The Newtonian liquid flow in a micromixer can be solved by the Navier–Stokes equation and the continuity equation [43]:

(1) $$\rho \left(\frac{\partial u}{\partial t}+\left(u\cdot \nabla \right)u\right)=\left(f-\nabla p+v\nabla^{2}u\right),$$

(2) ∇·u=0,

where u is the velocity vector, f is the body force, ρ is the density of water, p is the pressure, t is the time, and v is the dynamic viscosity of water.

The convection–diffusion equation is used to describe mixing in microchannels.

(3) $$\frac{\partial c}{\partial t}+\left(u\cdot \nabla \right)c=D\nabla^{2}c,$$

where c is the concentration and D is the diffusion coefficient of AA.

The mixing efficiency was calculated as follows [43]:

(4) $$M=1-\sqrt{\frac{1}{N}\overset{N}{\underset{i=1}{\sum }}\left(\frac{c_{i}-\bar{c}}{\bar{c}}\right)}$$

where N is the total number of sampling points, $c_{i}$ is the concentration at sampling point i, and $\bar{c}$ is the average concentration, M is the mixing efficiency, which has a value range from 0 to 1 for entirely unmixed fluids and mixed fluids. Here, M is the mixing efficiency, which is the mixing index of PBS and the sample at the outlet of the microchannel, ranging from 0 (unmixed) to 1 (complete mixed).

**Figure 6:**
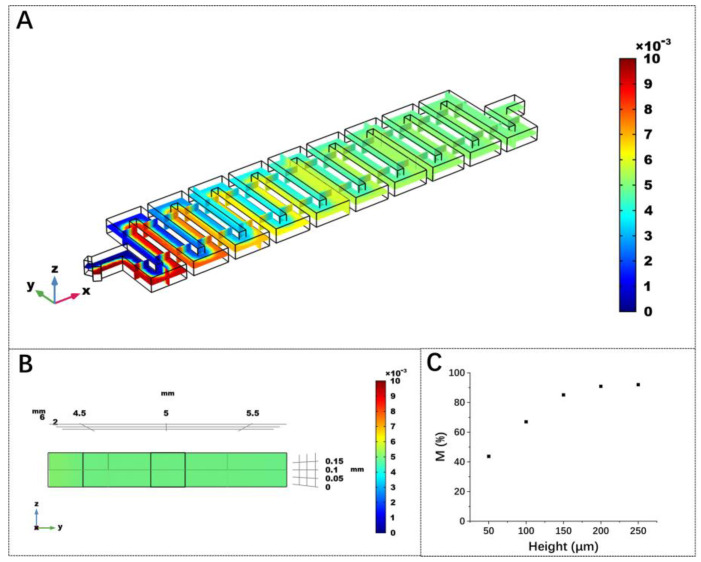
Numerical analysis of concentration distributions in the (**A**) *xy* plane of mixing channel and (**B**) *yz* plane of outlet; (**C**) effect of the microchannel height on the mixing efficiency.

The mixing efficiencies of straight channels and serpentine channels with heights of 50 µm, 100 µm, 150 µm, 200 µm and 250 µm were evaluated, respectively. The results show that when the inlet sample concentration is 100 µM, the samples can hardly mix in the straight channel, and the calculated mixing efficiencies at the outlets of the serpentine channels were 43.7%, 67.0%, 85.1%, 90.9% and 92.0% when the heights of the channels ranged from 50 µm to 250 µm (raw data shown in Supplementary Figures S2–S8, *n* = 10). Figure 6A,B show that the samples at the end of the serpentine channel have been well mixed. Figure 6C shows that when the channel height is below 200 µm, the mixing efficiency increases significantly with the increase in the height; when the channel height exceeds 200 µm, the improvement is not obvious. Considering the processing accuracy, the actual channel height was set to 200 µm.

The operation process of the finger-actuated micropump is as follows: firstly, press the actuation chamber while the same volume of sample and buffer are dripped onto the inlets separately; then, seal the vent before releasing the pressure; finally, release the actuation chamber, and the liquid from both inlets will be sucked into the mixing channel by the negative pressure inside and into the chamber due to the good elasticity of PDMS. A photograph of the experimental device is shown in Supplementary Figure S1. After experimental exploration, we finally set the height of the actuation chamber as 0.5–1 mm, and the wall thickness of PDMS as 1–2 mm. Here, 20 μL sample and 20 μL PBS buffer were pumped for the electrochemical detection experiments. It is also reliable to use only one inlet when the volume ratio of sample and buffer to be pumped is not 1:1 (adding the two types of liquid to one inlet while sealing the other one).

**Figure 7:**
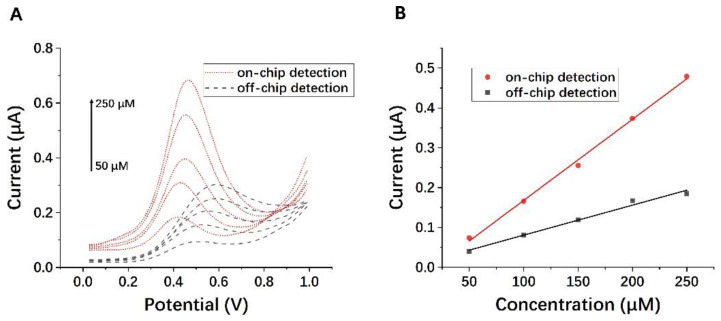
(**A**) DPV responses of on-chip detection and off-chip detection with increasing concentration of AA (bottom to top: 50 µM, 100 µM, 150 µM, 200 µM, 250 µM) (*n* = 3). (**B**) The calibration plot of current vs. concentration of on-chip detection and off-chip detection (*n* = 3).

We further compared the on-chip and off-chip electrochemical detection results, as shown in Figure 7. The sensitivity calculation formula of the sensor is as follows: sensitivity = mA^−1^, where m = the slope of the calibration plot, and A = the geometric surface area of the SPE. According to LOD = 3 S m^−1^, where S is the standard deviation of the response current, the signal-to-noise ratio = 3, and the measured limit of detection (LOD) is 1.038 μM, according to the calibration curve of *n* = 3. The LOD obtained by the DPV method is lower than that of the CV method. The linear equations of off-chip and on-chip detection were obtained successively:

Y_1_ = 0.0008X + 0.0188 (5)

R^2^ = 0.926, and a sensitivity of 0.027 A m^−1^cm^−2^.

Y_2_ = 0.0019X − 0.0381 (6)

R^2^ = 0.9941, and a sensitivity of 0.067 A m^−1^cm^−2^.

It is obvious from Figure 7A and Supplementary Table S1 that the signal intensity of on-chip detection was higher than that of off-chip detection. The calculated sensitivity of the former was 2.48 times that of the latter. The oxidation potential of on-chip detection was lower and the offset was smaller.

Moreover, due to the surface tension of aqueous solutions, it would be difficult for samples below 40 μL to completely cover the sensor surface for off-chip detection, whereas this can be easily achieved inside the chip because the liquid is confined to the microchamber at a specific size. As a result, on-chip testing not only has higher sensitivity but also requires a smaller volume of sample while eliminating the need to manually mix it.

A comparison of different AA electrochemical electrodes reported in the literature is listed in Table 1. It can be seen that sensors in this work exhibit a large linear range and good sensitivity.

### 3.3. Real Sample Analyses

We further used the sensor to detect actual samples (vitamin C tablets purchased from pharmacies), each of which was dissolved in PBS and diluted to 5000 μM, 500 μM and 50 μM. The calculated values of AA were 4867.5 μM, 472.8 μM and 48.9 μM (with 97.3%, 94.6% and 97.8% of recovery), respectively, which was reasonable considering the oxidation loss of AA during storage. Moreover, the additives in tablets (starch, dextrin, sodium carboxymethyl cellulose, CMC, CA, stearic acid, talcum powder, sorbitol, etc.) did not produce obvious interference signals. In addition, a commercially available vitamin beverage (Nongfu Spring, Hangzhou, China) was tested. Sample and PBS buffer were added to the chip in a volume ratio of 1:9. After conversion, the detected value was approximately 0.88 g/L, which was less than 10% different from the detected value of liquid chromatography and iodometry in a previous report [57]. This demonstrated the practicality and reliability of the device, indicating its potential to be applied to the POCT of tablets and beverages (raw data shown in Supplementary Figure S8).

## 4. Conclusions

In this work, a disposable, low-cost, screen-printed electrode is designed and manufactured, which achieves high sensitivity and rapid detection of AA, requiring only microliters of samples. After bonding with the microfluidic chamber, it can realize the rapid mixing of the sample and electrochemical detection. The on-chip detection shows better sensitivity and stability compared with the off-chip detection. The screen-printed electrodes are inexpensive consumables that could be produced in large quantities daily, and the 3D mold could be printed in 1 h with readily available commercialized resin materials, enabling quick and low-cost device manufacturing. The SPEs show good sensitivity and a linear correlation in PBS solution, and they have good selectivity in the presence of common interfering substances (Na^+^ ions, glucose, CA, etc.). Tests on commercial tablets and beverages indicate that they have broad application prospects in POCT.

## Figures and Tables

**Table 1:** Comparison of the analytical performance of different AA electrochemical sensors.

Electrode	Technology	Linear Range (µM)	Sensitivity (A M^−1^cm^−2^)	Reference
Fe_3_O_4_/r-GO/GCE ^1^	CV/DPV	160–7200	0.02	[53]
CuO-SPE ^2^	CV	100–8000	0.11	[54]
Au/RGO/GCE ^3^	CV/DPV	240–1500	-	[55]
[Ni(phen)_2_] ^2+^/SWCNTs/GCE ^4^	CV	30–1547	-	[56]
SPE	CV/DPV	10–5000	0.067	This work

^1^ Fe_3_O_4_ magnetic nanoparticle/reduced graphene oxide nanosheet-modified glassy carbon electrode; ^2^ CuO hemisphere screen-printed electrode; ^3^ Au nanoplate and reduced graphene oxide-modified glassy carbon electrode; ^4^ glassy carbon electrode modified with the nickel (II)bis(1,10-phenanthroline) complex and single-walled carbon nanotubes.

## Data Availability

Data available on request from the authors.

## Supplementary Materials

The following supporting information can be downloaded at: https://www.mdpi.com/article/10.3390/mi13091479/s1, Figure S1: Photograph of the device; Figures S2–S7: Numerical analysis; Figure S8: Detection of beverage; Figure S9: DPV detection in the presence of interfering substances; Table S1: On-chip and off-chip detection.
