# Supplementary figures and images for: Electrochemical Detection of Ascorbic Acid in Finger-Actuated Microfluidic Chip
Source: Micromachines (Basel). 2022 Sep 6;13(9):1479. doi: 10.3390/mi13091479 (PMC9502930; doi:10.3390/mi13091479)

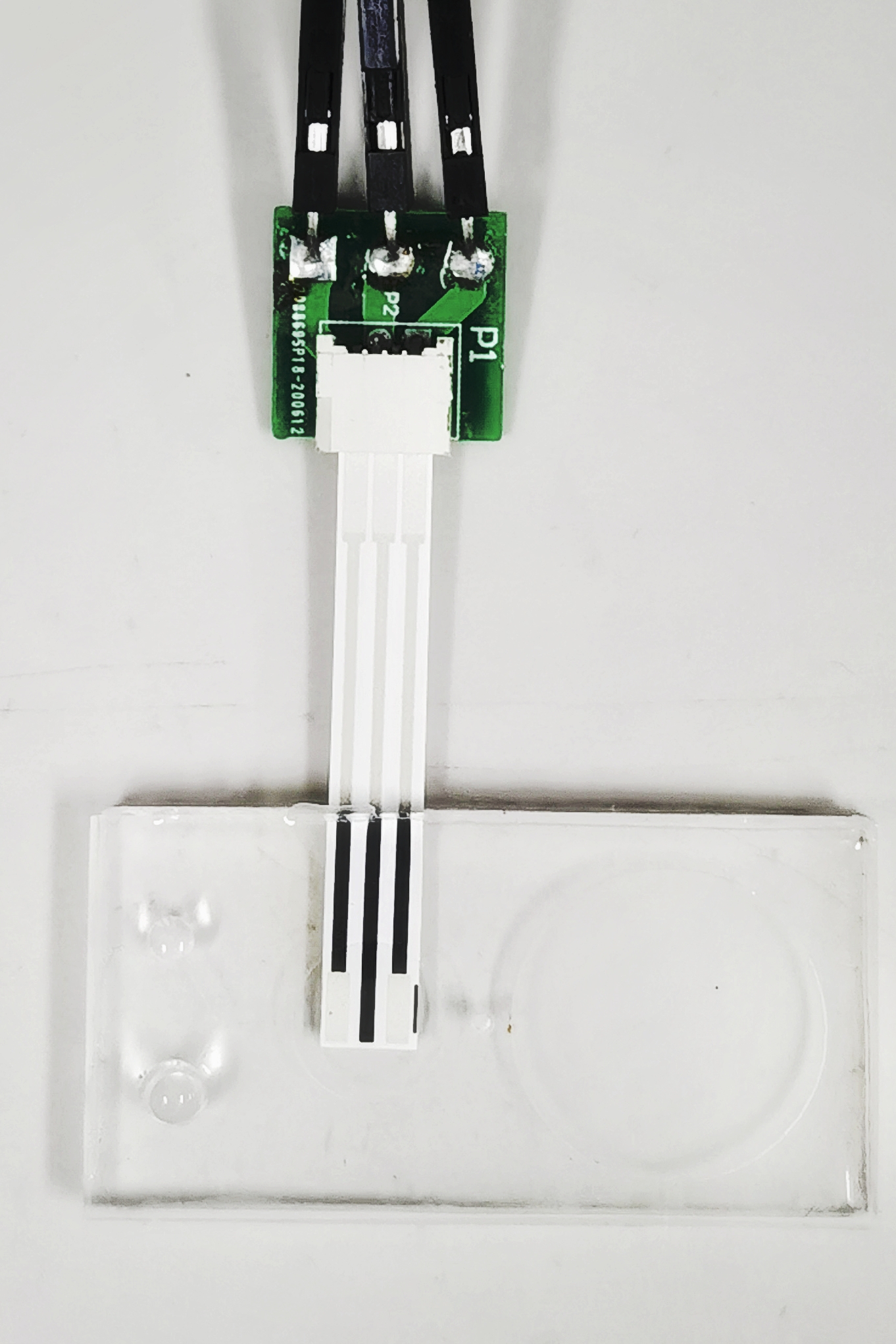

Supplement: Supplementary file 1 [file micromachines-13-01479-s001.zip › Figure S1.jpeg]

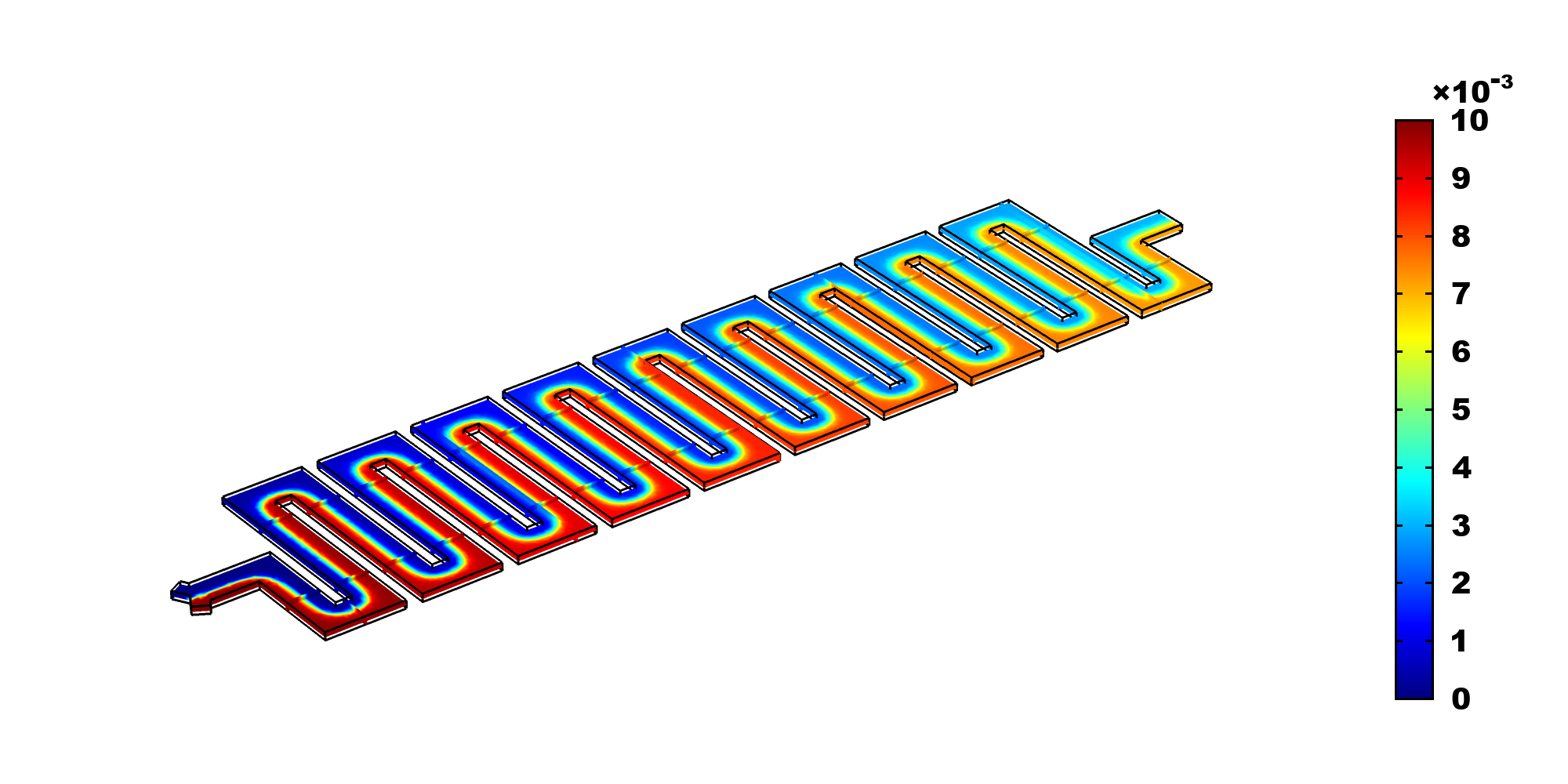

Supplement: Supplementary file 1 [file micromachines-13-01479-s001.zip › Figure S2-height-50.jpg]

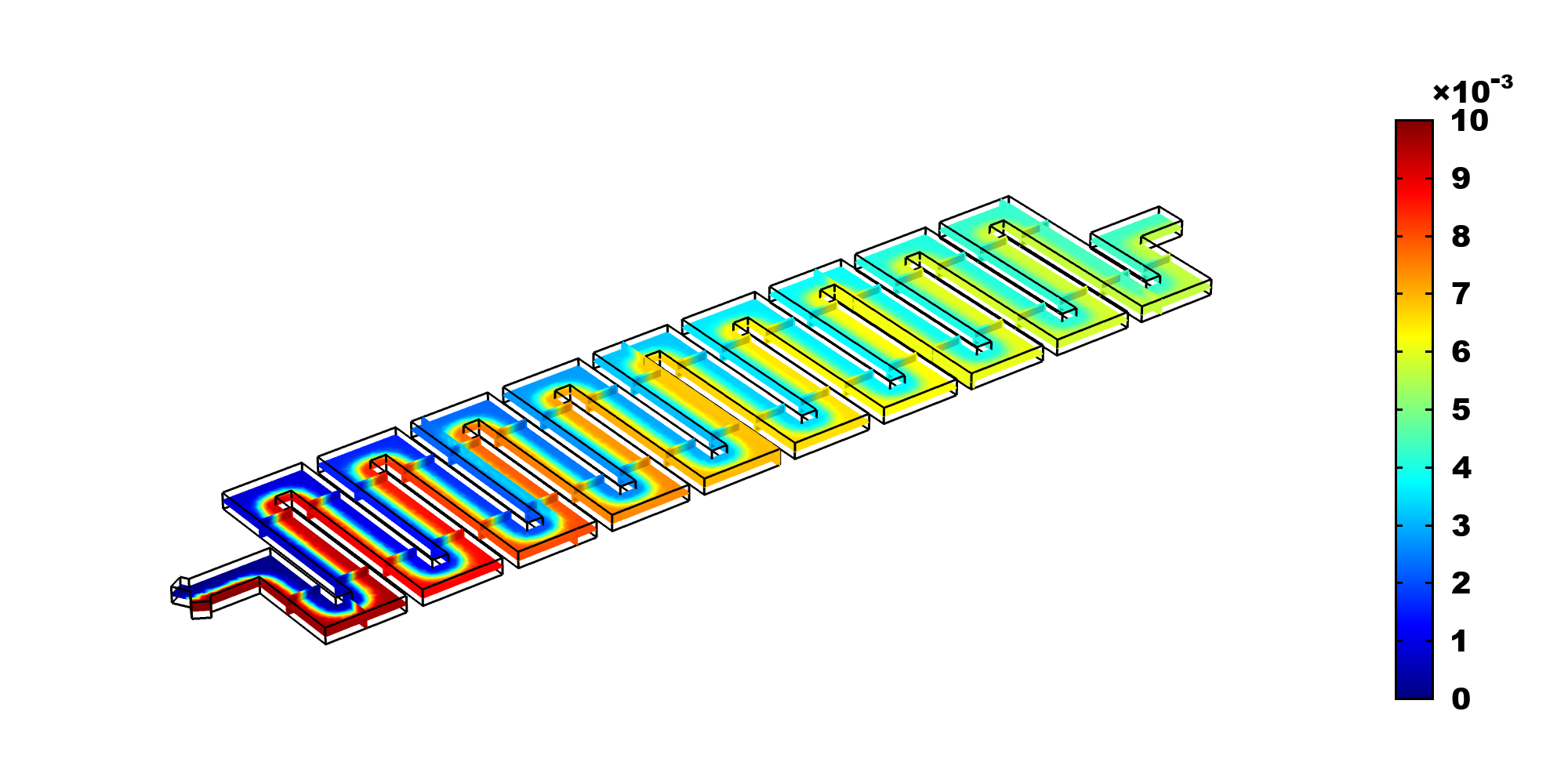

Supplement: Supplementary file 1 [file micromachines-13-01479-s001.zip › Figure S3-height-100.jpg]

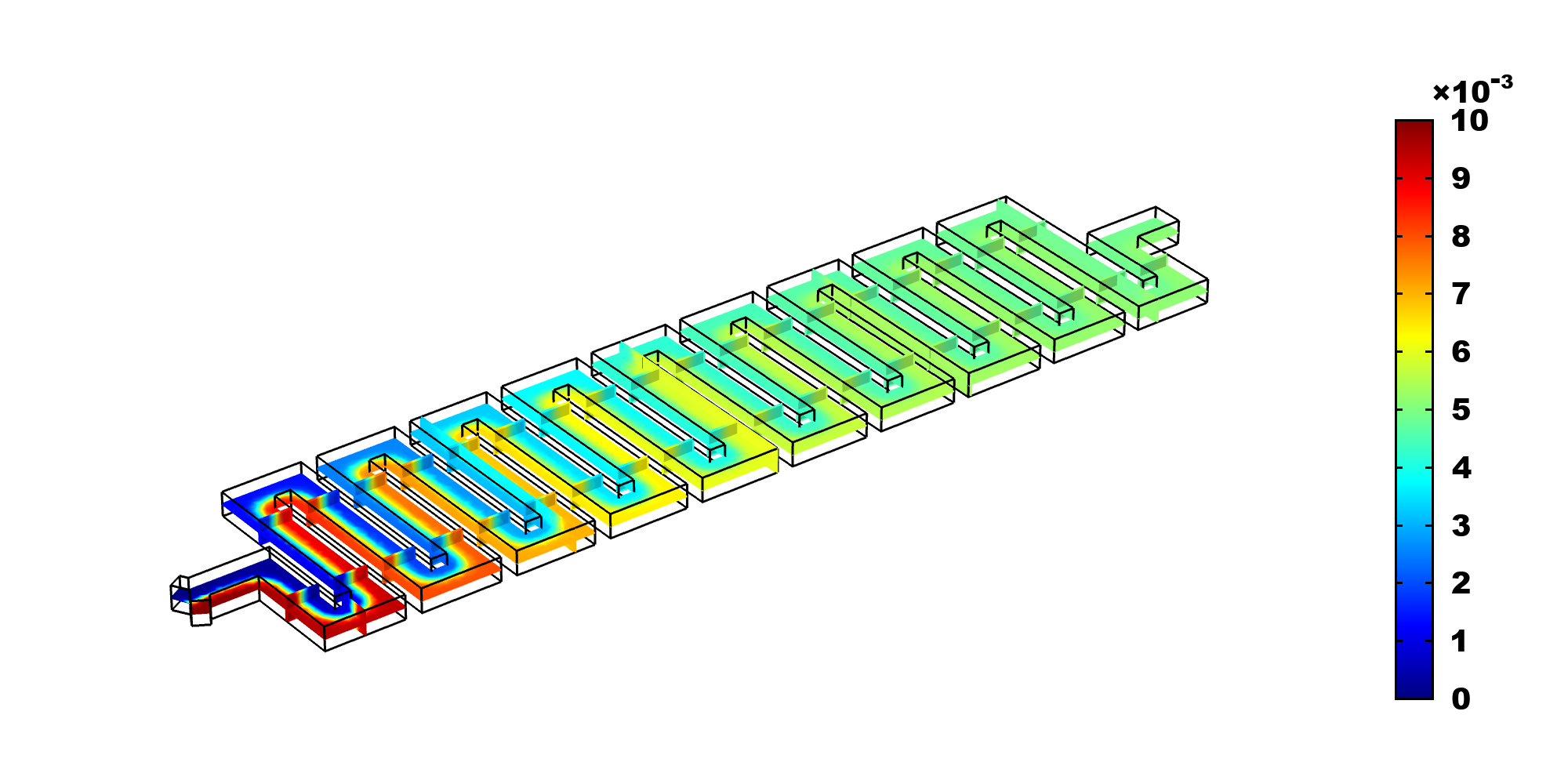

Supplement: Supplementary file 1 [file micromachines-13-01479-s001.zip › Figure S4-height-150.jpg]

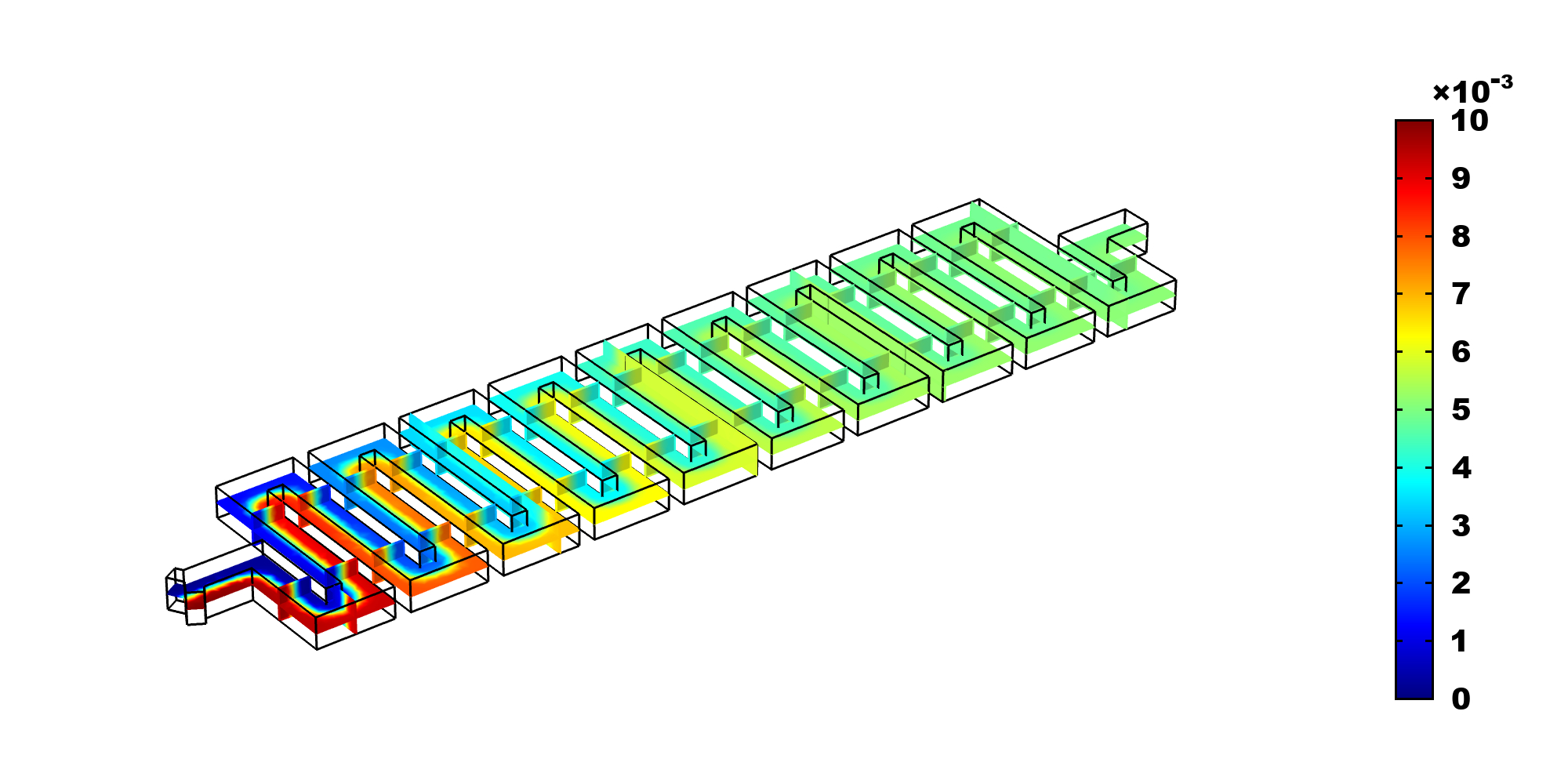

Supplement: Supplementary file 1 [file micromachines-13-01479-s001.zip › Figure S5-height-200.jpg]

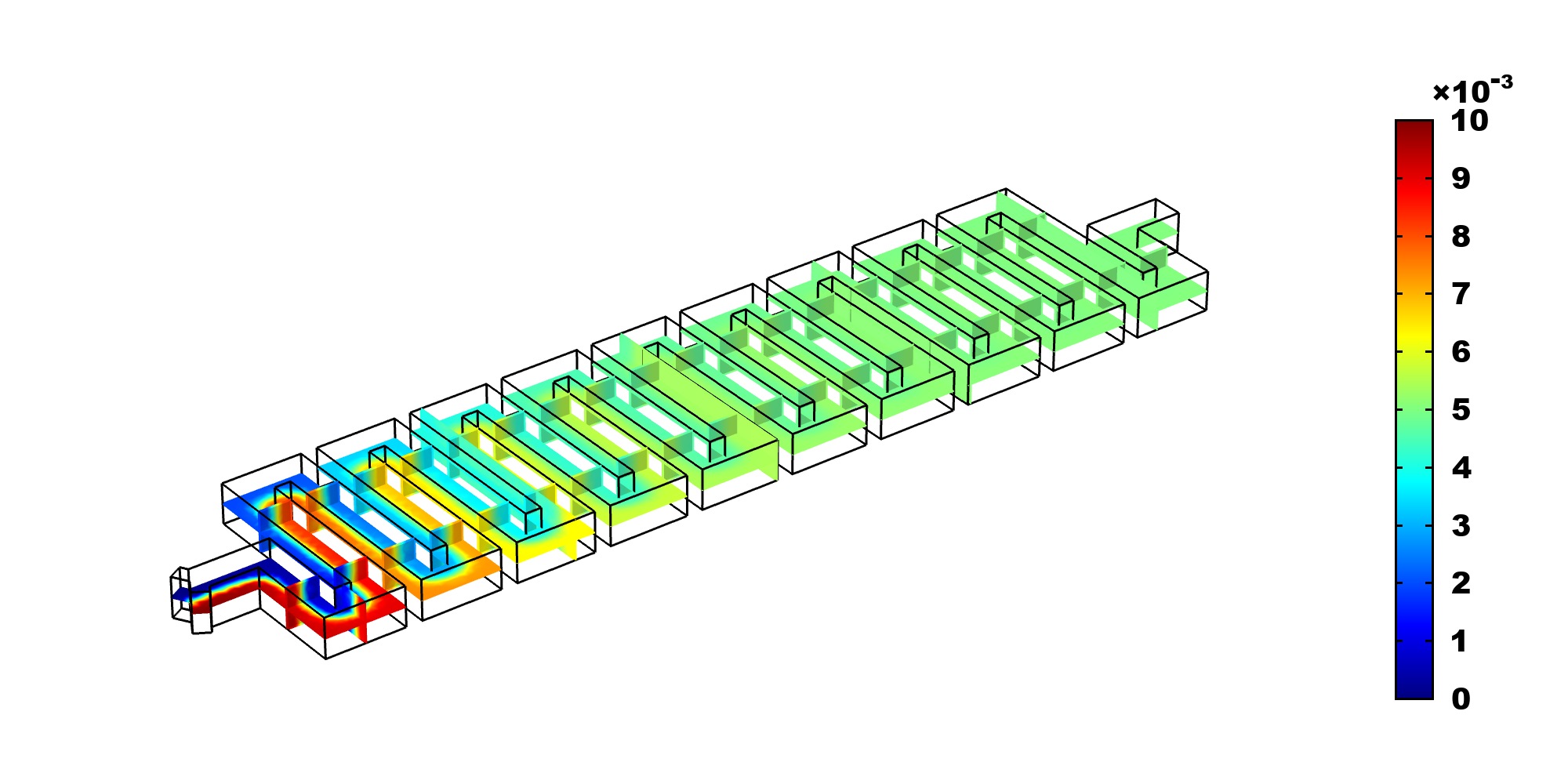

Supplement: Supplementary file 1 [file micromachines-13-01479-s001.zip › Figure S6-height-250.jpg]

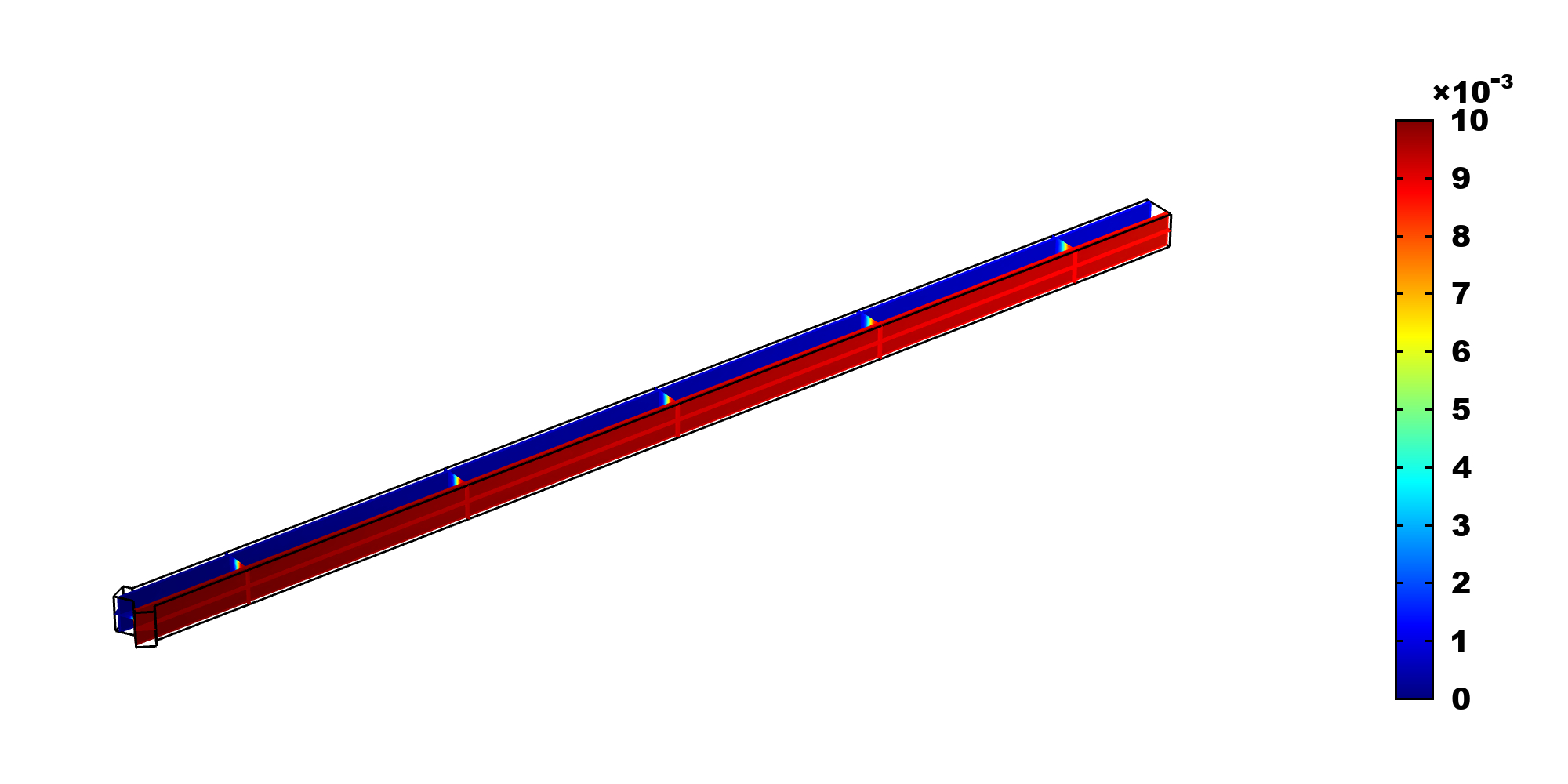

Supplement: Supplementary file 1 [file micromachines-13-01479-s001.zip › Figure S7-straight channal-1.jpg]

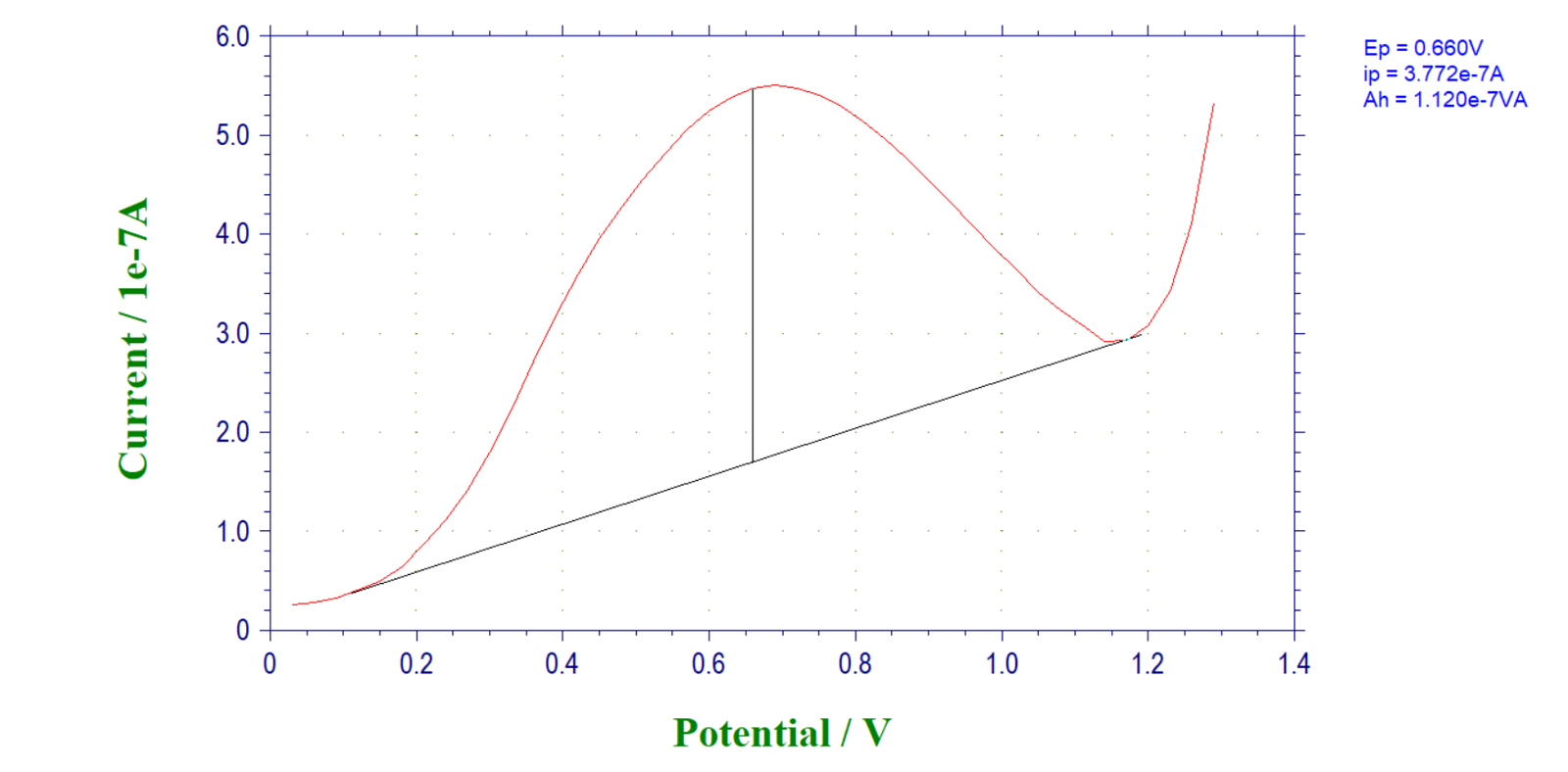

Supplement: Supplementary file 1 [file micromachines-13-01479-s001.zip › Figure S8-detection of beverage.jpg]

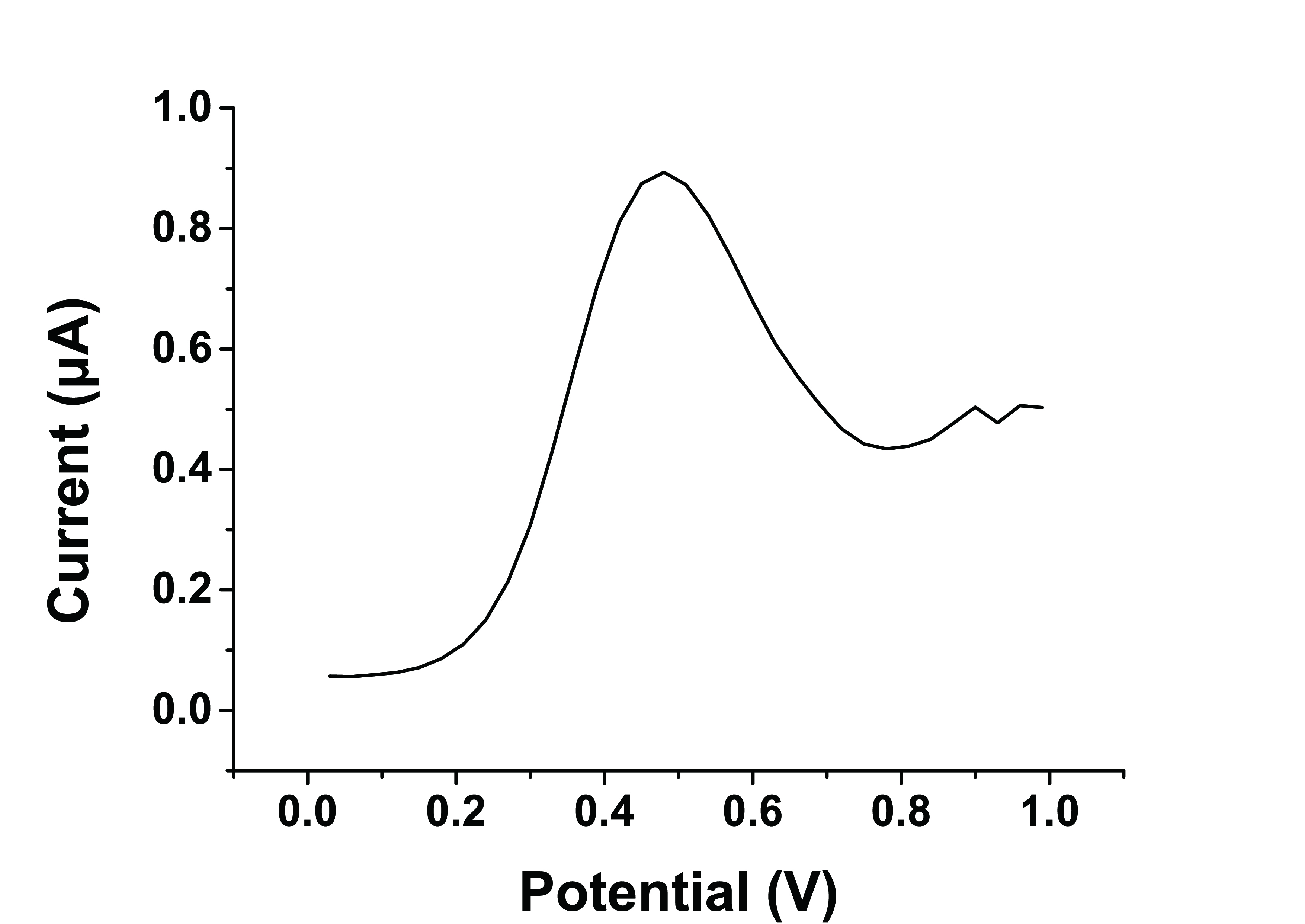

Supplement: Supplementary file 1 [file micromachines-13-01479-s001.zip › Figure S9-DPV detection in the presence of interfering substances.jpg]
